# The role of anti-inflammatory diets and supplementation in metabolic syndrome and symptom remission in adults with schizophrenia: a systematic review

**DOI:** 10.3389/fpsyt.2024.1506353

**Published:** 2025-01-07

**Authors:** Elizabeth Suschana, Thea Anderson, Catriona Hong, Arun Narikatte, Jillian Silverberg, Manu Suresh Sharma

**Affiliations:** ^1^ University of Connecticut School of Medicine, Farmington, CT, United States; ^2^ Frank H. Netter MD School of Medicine, Quinnipiac University, North Haven, CT, United States; ^3^ Library Services, University of Connecticut School of Medicine, Farmington, CT, United States; ^4^ Department of Psychiatry, Institute of Living, Hartford, CT, United States

**Keywords:** schizophrenia, anti-inflammatory diets, inflammation, vitamins, supplements, metabolic syndrome

## Abstract

**Introduction:**

Immune dysregulation and chronic inflammation have been hypothesized as potential pathways in metabolic syndrome and schizophrenia. Anti-inflammatory diets have the potential not only to treat metabolic syndrome but also to reduce the symptom burden in schizophrenia. The aim of this systematic review was to investigate the role of anti-inflammatory diets and vitamin supplementation in the management of metabolic syndrome and in symptom remission in people with schizophrenia.

**Methods:**

This systematic review included research articles from PubMed, EMBASE, Scopus, PsycINFO, and the Cochrane Central Register for Controlled Trials. The primary outcomes were markers of metabolic syndrome and symptoms of psychosis.

**Results:**

Our search identified 2,124 potential studies, of which 1,559 were screened based on the title and abstract, resulting in 81 full-text articles assessed for eligibility. A total of 17 studies were included, which demonstrated mixed findings on the impacts of anti-inflammatory diet interventions on metabolic markers and symptom remission in schizophrenia. Prebiotic, probiotic, and fish oil supplementation showed improvements in metabolic markers. Fish oil and vitamin D supplementation demonstrated symptom remission in some trials.

**Conclusion:**

It is important to consider that people with schizophrenia may experience common external barriers that hinder adherence to dietary interventions. These findings underscore the need for larger trials with standardized dietary protocols and consistent metabolic and symptom outcome measures in order to better understand the potential role of anti-inflammatory interventions in this population.

**Systematic review registration:**

https://www.crd.york.ac.uk/prospero/, identifier CRD42024511596.

## 1 Introduction

Lifetime history of schizophrenia spectrum disorders, including schizophrenia and schizoaffective and schizophreniform disorders, within the US is estimated at 1.8% (1). People with schizophrenia have an increased risk of premature mortality, with an estimated average potential life loss of 15–20 years (2). The earlier onset of serious medical diseases such as cardiovascular diseases, including coronary heart disease, is estimated to account for up to 80% of deaths within this population (2, 3). Metabolic syndrome has been identified as a patient factor contributing to the increased risk of cardiovascular disease and, ultimately, to premature mortality (2). Recent estimates of people with schizophrenia who met the criteria for metabolic syndrome have been as high as 60%, compared to 30% within the general population (4). The contributing factors to the development of metabolic syndrome include the side effects of antipsychotics and their effects on metabolism, comorbid substance use, lifestyle factors, adverse diets and poor nutrition, and negative symptoms (2).

A systematic review and meta-analysis examining the global prevalence of metabolic syndrome in patients with schizophrenia found a pooled prevalence of 41.3%, with significant variability across regions, ranging from 79.1% in France to 18.03% in China (5). This high prevalence, affecting over one-third of patients with schizophrenia, underscores the critical need for targeted interventions to reduce the associated chronic disease burden and mortality, reinforcing the importance of this study’s focus on the management of metabolic syndrome within this vulnerable population (5). Individuals with schizophrenia face disproportionately high rates of medical comorbidities, with metabolic syndrome present in approximately 32.5% of cases, which is largely influenced by factors such as illness duration and specific antipsychotic medications, particularly clozapine (6). People with severe mental illness (SMI), such as schizophrenia and bipolar disorder, face a 20-year reduction in life expectancy, primarily due to preventable cardiometabolic diseases—a disparity that appears to be worsening (7). While multidisciplinary teams provide varied treatments, lifestyle interventions to improve physical health have historically been lacking (8).

Metabolic syndrome and antipsychotic-induced weight gain negatively impact quality of life and are common reasons for medication non-adherence and premature discontinuation, creating barriers to symptom remission among people with schizophrenia (9–11). Metabolic syndrome is defined as a group of conditions including obesity, high blood pressure, high blood sugar, high triglycerides, and low high-density lipoprotein (HDL) cholesterol (12).

People with schizophrenia and comorbid metabolic syndrome have poorer cognitive performance, possibly resulting in worse symptom remission (13, 14). Medications such as metformin are used in weight reduction due to antipsychotic therapy (11, 15). Lifestyle counseling and exercise interventions have been shown to be more effective for weight reduction compared with low-metabolic-risk antipsychotics and metformin (15). Although lifestyle interventions and dietary modifications are effective methods to reduce metabolic syndrome, no clear consensus exists on the most effective dietary approaches for people with schizophrenia.

Immune dysregulation and chronic inflammation have been hypothesized as potential risk factors for both metabolic syndrome and the development and worsening of symptoms in individuals with schizophrenia. The complement system, particularly C1q and C3, plays a crucial role in both the immune response and the regulation of synaptic pruning. Synaptic pruning, which occurs during a critical period between adolescence and early adulthood, coincides with the typical onset of psychosis symptoms in schizophrenia (16). PET scans of the prefrontal cortex have revealed a reduction in dendritic spine density during this time (16, 17).

Astrocytes upregulate C1q and C3, facilitating opsonization and the targeted destruction of synapses by the microglia (17). Individuals with schizophrenia exhibit elevated pro-inflammatory cytokines (e.g., IL-1β, IL-6, IL-8, and TNF-α) released by the microglia (18, 19). These cytokines affect the downstream neurotransmission pathways, notably increasing the level of kynurenic acid in the kynurenine pathway, which disrupts the neurotransmitter balance and further amplifies inflammation through increased cytokine production (20).

In addition, an imbalance between the production of reactive oxygen species and antioxidants leads to increased oxidative damage (21). Patients with schizophrenia have been shown to have lower levels of the antioxidant glutathione in the medial prefrontal cortex and the striatum, areas that are both implicated in the pathophysiology of psychotic symptoms (21). Oxidative stress also negatively affects neurodevelopment in patients with schizophrenia via dysfunction of the *N*-methyl d-aspartate (NMDA) receptors in the process of synaptic plasticity (21). Previous studies have demonstrated the potential of antioxidant supplements such as omega-3 polyunsaturated fatty acids and vitamin D as adjunctive treatments in reducing the symptoms of schizophrenia (21). However, these studies focusing on dietary supplements have not directly looked at improvements in the metabolic markers.

Individuals with schizophrenia frequently have adverse, nutrient-poor diets, which include a higher intake of animal fats compared with vegetable fats, a lower intake of fruits, and a higher consumption of instant meals (22–24). Excessive saturated fat intake and low fiber and fruit intake result in higher levels of the inflammatory markers, including tumor necrosis factor (TNF), C-reactive protein (CRP), and interleukin 6 (IL-6), which have been implicated as a possible pathophysiology in schizophrenia development and symptom exacerbation (25). There is evidence that specific anti-inflammatory dietary regimens such as the Mediterranean diet can lead to a significant reduction of these inflammatory markers while also improving metabolic outcomes such body weight and lipids (26). The potential anti-inflammatory nutritional avenues identified are omega-3 fatty acids, vitamin C, vitamin D, resveratrol, Mediterranean diet, ketogenic diet, and dietary approaches to stop hypertension (DASH) (25).

Ultimately, these processes lead to increased neuroinflammation. This contributes to dysregulation in neurotransmission systems, including alterations in the dopamine pathway, a key factor in schizophrenia symptoms and neurodevelopment (20). There is a potential for specialized diets and vitamin supplements to not only improve the metabolic outcomes but also help reduce inflammation, thereby bringing about an improvement in the symptoms of psychosis. The aim of this review was to investigate the possible role of anti-inflammatory diets and vitamin supplementation in addressing both symptom remission and metabolic syndrome in people with schizophrenia, schizoaffective disorder, and first-episode psychosis.

## 2 Methods and analysis

### 2.1 Objectives

The aims of this systematic review were as follows:

1. To evaluate the effectiveness of anti-inflammatory diets in the treatment of metabolic syndrome in patients with psychosis;
2. To assess the methodological quality and the strength of evidence regarding anti-inflammatory diets in patients with psychosis, specifically for the treatment of metabolic syndrome and for symptom remission; and
3. To determine the appropriate dietary interventions that enhance symptom control for patients with psychosis.

### 2.2 Protocol and registration

**Figure 1:**
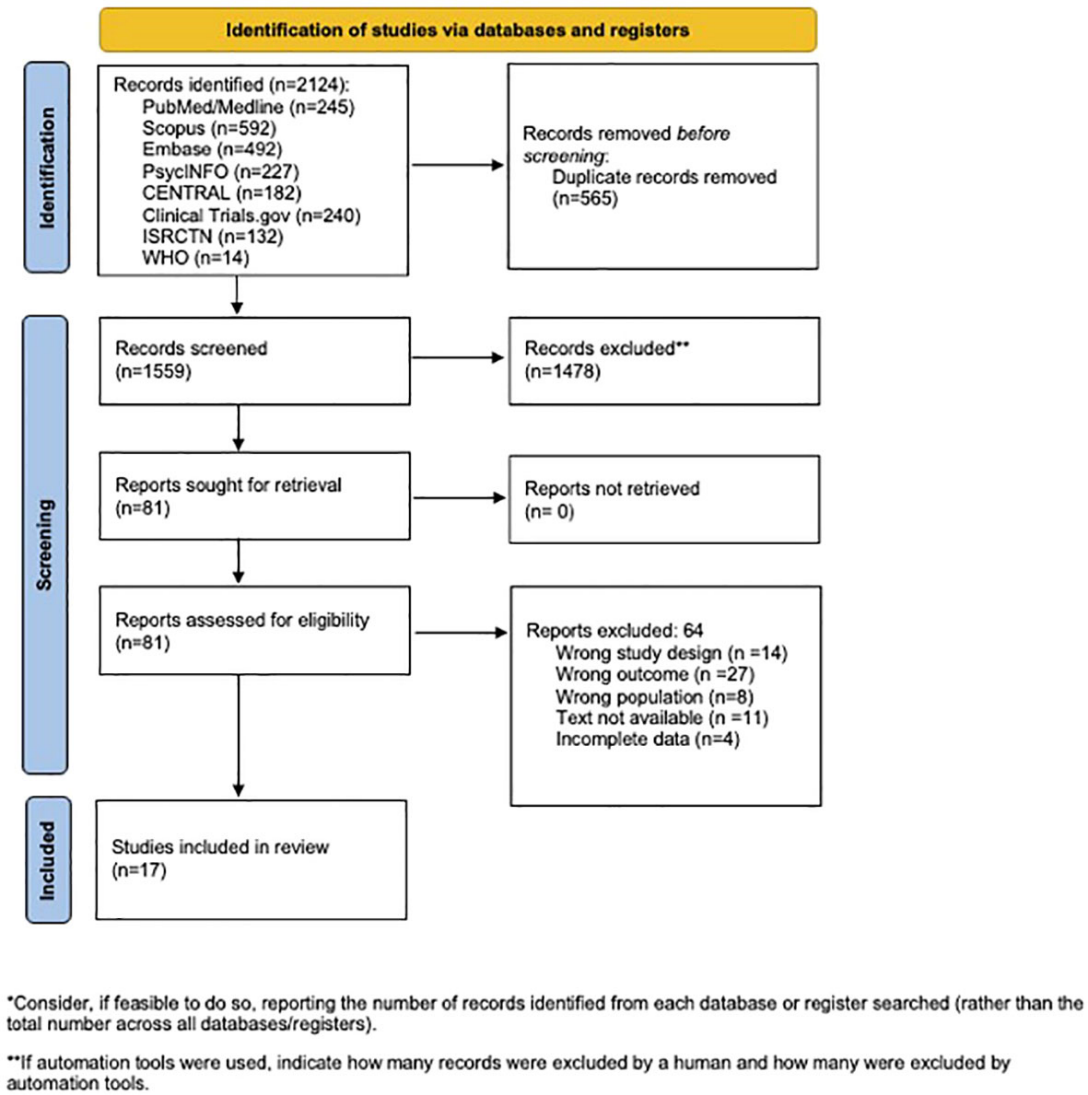
Preferred Reporting Items for Systematic Reviews and Meta-Analyses (PRISMA) flow diagram.

This systematic review was conducted in adherence to the Preferred Reporting Items for Systematic Reviews and Meta-Analyses (PRISMA) guidelines (**Figure 1**) (27). The protocol was registered in the International Prospective Register for Systematic Reviews, PROSPERO (protocol no. CRD42024511596).

### 2.3 Outcome measures

The primary outcomes included the following measures:

1. Changes in the metabolic profile as measured by body mass index (BMI), blood glucose, lipids, blood pressure, weight, and waist circumference, among others.
2. Changes in the symptoms of psychosis measured using a standardized instrument, such as the Positive and Negative Symptom Scale for Schizophrenia (PANSS), the Scale for the Assessment of Negative Symptoms (SANS), and the Scale for the Assessment of Positive Symptoms (SAPS).

### 2.4 Eligibility criteria

The eligibility criteria for the participants, intervention, comparison, outcome, and study design (PICOS) domains are presented in **Table 1**.

**Table 1:** Description of the population, intervention, comparison, outcome, and study design domains.

Domain	Inclusion criteria	Exclusion criteria
Population	Patients with schizophrenia, schizoaffective disorder, and first episode psychosis without age or gender predilection	Participants without psychotic disorder; medication-induced psychosis
Intervention	Anti-inflammatory diets or vitamins	Non-anti-inflammatory diets or vitamin
Comparator	Placebo or treatment as usual	
Outcome	*Primary outcomes*: Changes in the metabolic profile Changes in the symptoms of psychosis	
Study design	Randomized controlled trials, non-randomized controlled trials, retrospective studies, and cohort studies	Animal studies, case reports, case series, reviews, letters, commentary/editorial articles, and clinical trials with no results

### 2.5 Information sources and search strategy

The search strategies were developed by an experienced health sciences librarian and other members of the research team. The strategy was translated for the other included databases. The librarian searched the following databases and trial registries from the date of inception through to October 24, 2024: PubMed (including pre-MEDLINE and non-MEDLINE; from 1945 to October 2024), EMBASE (Elsevier; from 1974 to October 2024), Scopus (Elsevier; from 1966 to October 2024), PsycINFO (EBSCO, from 1887 to October 2024), and Cochrane Central Register for Controlled Trials (Wiley, through to October 2024). Unpublished literature was also considered in this review through a search of the International Clinical Trials Registry Platform, the International Standard Registered Clinical/Social Study Number, and ClinicalTrials.gov. Unpublished studies with no results were not included in our analysis. A number of items found in CENTRAL fell into this category as well (see **Supplementary Table 1** for the search strategy). The Boolean operator NOT, along with the database limiters, was applied when appropriate to omit items deemed undesirable to the project (see above for the exclusion criteria). The citation management tool EndNote (Clarivate, Philadelphia, PA) was used to de-duplicate and to manage all citations.

### 2.6 Data extraction and coding

Four reviewers independently extracted data (**Table 2**). Specific data including the means and *p*-values were recorded as feasible. In the event of missing or ambiguous data in the included studies, the researchers made efforts to contact the corresponding authors. If missing data could not be obtained despite these attempts, the study was excluded from the analysis. Discrepancies were resolved via discussion until a consensus was reached or by a third reviewer. The following data were recorded.

1. Basic information of the study: first author, year, and country of publication.
2. Study characteristics: study inclusion criteria, participant details (i.e., age, gender, diagnosis, baseline symptom severity, and baseline metabolic markers).
3. Details of the study intervention(s) and control intervention(s).
4. Results.
5. Risk of bias assessment.

**Table 2:** Description of the study characteristics, limitations, and the bias assessment score (BAS).

Study	Study design	Population	Intervention			Limitations	BAS
			Duration	Experimental	Control		
Deutsch et al. (28)	RCT	18- to 70-year-olds with schizophrenia or schizoaffective disorder, on a stable second-generation antipsychotic for at least 4 weeks, and at least moderately severe symptoms on PANSS^a^	3.5 months	*n* = 37 2,000 mg/day of galantamine/CP-coline	*n* = 37 Placebo supplementation	1) Small sample size 2) Inclusion of confounding variables such as smoking	2/3
Dickerson et al. (29)	RCT	18- to 65-year-olds with schizophrenia, at least moderately severe psychotic symptoms on PANSS, and compliance with maintenance antipsychotic medication^a^	3.5 months	*n* = 33 1 probiotic tablet daily	*n* = 32 Not described	1) Small sample size 2) No description on the placebo group 3) No description of the blinding	2/3
Fenton et al. (30)	RCT	18- to 65-year-olds with schizophrenia with no medication changes in the prior 30 days, on medication, and with presence of significant residual symptoms on PANSS^a^	4 months	*n* = 43 3 g of omega-3 fatty acid daily	*n* = 44 Mineral oil placebo pill	1) Small sample size	2/3
Jamilian et al. (31)	RCT	15- to 55-year-olds with schizophrenia^a^	2 months	*n* = 30 1,000 mg omega-3	*n* = 30 Placebo supplementation	1) Small sample size 2) Short duration of study	2/3
Kelly et al. (32)	RFS	Diagnosis of schizophrenia or schizoaffective disorder, on a stable medication dose for at least 4 weeks, and positive AGA IgG screening^a^	1.25 months	*n* = 9 10 g of rice flour (gluten-free diet) in a protein shake and standardized gluten-free diet	*n* = 9 10 g of gluten four in a protein shake and standardized gluten-free diet	1) Small sample size 2) Short duration	2/3
Kelly et al. (33)	PCT^E^	Inpatients aged 16–64 years with schizophrenia and with no antipsychotic changes in the past 14 days^a^	2 weeks	*n* = 5 4 g of oligofructose-enriched inulin three times daily	None	1) Small sample size 2) Open-label design	0/3
Roffman et al. (34)	RCT	18- to 68-year-olds with schizophrenia with psychiatric stability and persistent symptoms despite at least 6 months of antipsychotic treatment, including 6 weeks at a stable dose, and a PANSS score of 60 or more^a^	4 months	*n* = 94 2 mg folic acid and 400 µg vitamin B12	*n* = 46 Placebo supplementation	1) Focused on European ancestry 2) Small sample size	3/3
Fadai et al. (35)	RCT^B^	Male patients 18–65 years old with schizophrenia and on a stable olanzapine regimen with no evidence of metabolic syndrome^b^	3 months	Arm 1: *n* = 20 15 mg of saffron twice daily Arm 2: *n* = 20 15 mg of crocin twice daily	*n* = 21 Not described	1) Small sample size 2) Only male patients included 3) No inclusion criteria for baseline symptom severity	3/3
Neriman et al. (36)	Quasi-experimental	18 – 65 years old with schizophrenia and had stable medication regimens and low 25-OHD levels^b^	4 months	*n* = 40 Oral vitamin D supplementation until sufficient levels are reached	None	1) Small sample size 2) Only included participants with low vitamin D levels 3) No control group	***
Pawelckyz et al. (37)	RCT	16- to 35-year-olds with first episode of schizophrenia and currently psychotic^a, c^	2–3 months	*n* = 36 2.2 g fish oil	*n* = 35 Olive oil	1) Small sample size 2) Only included first episode of schizophrenia 3) Limited age range	2/3
Qiao et al. (38)	RCT	Inpatients 18–60 years old with schizophrenia, a PANSS >50, violent behavior (MOAS score >4), and elevated aggression scores while on antipsychotics^a, c^	3 months	*n* = 28 900 mg fish oil	*n* = 22 Vitamin E	1) Small sample size 2) Short intervention period 3) Variable intervention periods between participants depending on discharge from the inpatient unit	2/3
Qiao et al. (39)	RCT	Acutely hospitalized with schizophrenia and demonstrating violent behavior	2 months	*n* = 32 900 mg fish oil	*n* = 35 10 mg vitamin E	1) Small sample size 2) Gender imbalance 3) Placebo was vitamin E, which may be an “active” compound	2/3
Rensburg et al. (40)	RCT	18- to 65-year-olds with schizophrenia and persistent symptoms (PANSS score >50) while on fixed doses of antipsychotics at least 6 months prior to the trial	3 months	*n* = 16 3 g ethyl-EPA daily	*n* = 16 Placebo capsule	1) Variable dietary intake 2) Small sample 3) Short intervention	1/3
Sevillano-Jimenez et al. (41)	RCT	18- to 65-year-olds with schizophrenia and clinical stability 6 months prior to the initiation of the trail	6 months	*n* = 23 Dietary counseling to increase prebiotic and probiotic intake	*n* = 21 Conventional dietary advice	1) Assessment of mainly parametrics of dietary modification, not outcomes 2) During COVID-19 pandemic, leading to variability 3) High dropout rate in the intervention group	3/3
Soric et al. (42)	RCT	Hospitalized patients 18–67 years old with schizophrenia and clinical stability	3 months	*n* = 33 DASH diet with caloric restriction of 400 kcal/day	*n* = 34 Normal hospital diet	1) Short time 2) Lack of standardized diet as participants could purchase outside food	2/3
Vaughan and McConaghy (43)	RCT	Outpatient patients with schizophrenia	5 months	*n* = 11 Megavitamin	*n* = 9 25 mg vitamin C	1) Small sample size 2) Placebo was a vitamin supplementation	3/3
Zhang et al. (44)	RCT	19- to 65-year-olds with schizophrenia, serum total cholesterol (TC) ≥6.22 mmol/L or serum triglycerides (TG) ≥2.26 mmol/L	1 month	*n* = 39 Konjac flour 1,800 kcal diet for women 2,200 kcal diet for men	*n* = 37 Maltodextrin supplementation 1,800 kcal diet for women 2,200 kcal diet for men	1) Short intervention 2) Small sample size 3) Inadequate sample size for subgroup analysis	3/3

*BAS*, bias assessment score; *RCT*, randomized controlled trial; *PCT*, pilot clinical trial; *RFS*, randomized feasibility trial; *DASH*, dietary approaches to stop hypertension.

a In these studies, the participants were diagnosed using the DSM-V criteria.

b In these studies marked, the participants were diagnosed using the DSM-IV criteria.

c In these studies marked, the participants were diagnosed using ICD-10 diagnostic criteria.

### 2.7 Bias assessment

All reviewers independently evaluated the risk of bias of the included studies. Each study was rated using a validated three-point questionnaire that included randomization, double blinding, and the withdrawal and dropout descriptions (**Table 3**) (45). A score of 1 out of 3 was defined as high risk of bias. A score of greater than 2 out of 3 was defined as low risk of bias.

**Table 3:** Description of the risk bias assessment and scoring via a validated questionnaire.

Risk of bias assessment validated questionnaire
Studies were assigned 1 point for yes or 0 point for no in the following categories. The maximum score is 3 points. 3 points = low risk of bias 2 points = moderate risk of bias 1 point = high risk of bias
*Randomization*: 1 point was given for description of appropriate randomization (table of random numbers, computer generated, etc.). A point of 0 was given if there was no description of randomization or the randomization was not appropriate (date of birth, date of admission, hospital number, or alternation).
*Double blinding*: 1 point was given if the word “double blind” was used or the method of blinding was appropriate, including neither the study personnel nor the participants would identify the intervention or statements such as active placebos, identical placebos, or dummies were mentioned.
*Withdrawals and dropouts*: 1 point was given for a description of the dropouts and withdrawals, defined as participants who did not complete the observation period or who were not included.

## 3 Results

Our search identified 2,124 potential studies, of which 1,559 were screened based on the title and abstract, resulting in 81 full-text articles assessed for eligibility. Ultimately, 17 studies met the inclusion criteria and were included in the analysis (**Table 2**). Most of the studies were randomized controlled trials (RCTs) conducted in adults with schizophrenia aged 18–65 years in an outpatient setting, with sample sizes ranging from 5 to 80. The duration of intervention ranged from 2 weeks to 6 months, with most interventions lasting 3 months or more. Only three studies included dietary interventions, such as a gluten-free diet or the DASH diet. Most interventions were of vitamin or mineral supplementation of various doses. The most common vitamin supplementation across the studies was fish oil or omega-3s.

Most of the studies did not assess metabolic markers. One study that looked at prebiotic and probiotic intake found a significant reduction in BMI (*p* < 0.001) and waist circumference (*p* < 0.001) between the control and experimental groups (**Table 4**). In one study, fish oil supplementation resulted in a significant difference in the levels of triglycerides (*p* = 0.094), fasting glucose (*p* = 0.045), and low-density lipoprotein (LDL) cholesterol (*p* = 0.094) between the control and experimental groups (**Table 4**). Another study compared crocin and saffron supplementation to a control group and noted a significant difference in the fasting glucose level (*p* = 0.004), but no other metabolic markers (**Table 4**).

**Table 4:** Description of the changes in metabolic markers.

Study	Metabolic markers					
	BMI	WC	TG	FBG	LDL	SBP
Fadai et al. (35) Saffron, crocin	–	At 3 months: -Saffron: 92.1 -Crocin: 91.9 -Control: 98.4 *p* = 0.19	At 3 months: -Saffron: 85 -Crocin: 102.8 -Control: 102.8 *p* = 0.723	At 3months: -Saffron: 100.5 -Crocin: 97.9 -Control: 108.6 *p* = 0.004*	At 3 months: -Saffron: 168.5 -Crocin: 178.5 -Control: 181.7 *p* = 0.598	At 3 months: -Saffron: 116.5 -Crocin: 109.7 -Control: 115.9 *p* = 0.065
Kelly et al. (32) Gluten-free diet	–	–	No change	Cohen’s *d* = −0.36	–	–
Pawelckyz et al. (37) Fish oil	–	At 2 months, mean change: -Control: 4.32 -Experimental: 3.99 Mean difference = −0.33 *p* = 0.819	At 2 months, mean change: -Control: 8.48 -Experimental: −16.02 Mean difference = −24.5 *p* = 0.094	At 2 months, mean change: -Control: 10.55 -Experimental: 3.55 Mean difference = −7.0 *p* = 0.045*	At 2 months, mean change: -Control: 16.37 -Experimental: 2.52 Mean difference = −13.85 *p* = 0.094	At 2 months, mean change: -Control: 4.07 -Experimental: 0.68 Mean difference = −3.39 *p* = 0.1576
Sevillano-Jimenez et al. (41) Pre-/probiotic	Control: -Pre: 27.5 -Post: 27.2 *p* = 0.323 Experimental: -Pre: 29.5 -Post: 27.9 *p* < 0.001*	Control -Pre: 97.6 -Post: 101.2 *p* = 0.322 Experimental -Pre: 105.7 -Post: 102.1 *p* < 0.001*	–	–	–	–
Soric et al. (42) DASH and calorie restriction	Control: -Pre: 27.47 -Post: 26.96 Experimental: -Pre: 28.95 -Post: 28.16 *p* = 0.281	Control: -Pre: 106.92 -Post: 104.47 Experimental: -Pre: 109.09 -Post: 105.55 *p* = 0.690	Control: -Pre: 1.81 -Post: 1.83 Experimental: -Pre: 2.15 -Post: 2.32 *p* = 0.056	–	Control: -Pre: 3.07 mmol/L -Post: 3.27 mmol/L Experimental: -Pre: 3.19 mmol/L -Post: 3.04 mmol/L *p* = 0.255	Control: -Pre: 129.41 -Post: 126.25 Experimental: -Pre: 130.38 -Post: 126.52
Zhang et al. (44) Konjac flour and calorie restriction	Non-significant increase in BMI in both the control and experimental groups	–	Non-significant decrease in the experimental group	Overall: Non-significant change Women: Statistically significant decrease in FBG	Total cholesterol: decreased significantly. −3.90 ± 14.38 *p* = 0.005 (overall) HDL and LDL change not significant	Non-significant increase in SBP in both the control and experimental groups

BMI, body mass index; *WC*, waist circumference; *TG*, triglycerides; *FBG*, fasting blood glucose; *LDL*, low-density lipoprotein; *SBP*, systolic blood pressure; *HDL*, high-density lipoprotein. *Indicates that the p-value was statistically significant.

All but four studies assessed the symptoms of schizophrenia, including the total symptom scores, the positive symptom scores, and the negative symptom scores, using various validated symptom scales (**Table 5**). The symptom scales used included the Positive and Negative Syndrome Scale (PANSS), the Brief Psychiatric Rating Scale (BPRS), the Scale for the Assessment of Negative Symptoms (SANS), the Scale for the Assessment of Positive Symptoms (SAPS), Beck’s Depression Inventory (BDI), and Brief Symptom Inventory (BSI). Supplementation with fish oil and vitamin D led to significant improvements in the total PANSS scores, positive symptom scores, and negative symptom scores in a few included studies, but insignificant improvements in others (**Table 5**).

**Table 5:** Description of the changes in the psychiatric symptom scale scores.

Study	Schizophrenia symptoms		
	Total symptom score	Positive symptoms	Negative symptoms
Deutsch (28) Galantamine/CP-choline	No significant difference between groups	No significant difference between groups	No significant difference between groups
Dickerson et al. (29) Probiotic	No significant difference between groups	No significant difference between groups	No significant difference between groups
Fenton et al. (30) Omega-3 fatty acids	Total Positive and Negative Syndrome Scale: -Control: 18 -Experimental: 16 Mean difference = −2.0 *p* = 0.84	–	–
Jamilian et al. (46) Omega-3 fatty acid	Total score on PANNS: -Control: 52.43 -Experimental: 49.13 Mean difference = −3.3 *p* < 0.05*	PANSS: -Control: 14.66 -Experimental: 14.00 Mean difference = −2.66 *p* > 0.05	PANSS: -Control: 11.26 -Experimental: 12.13 Mean difference = 0.87 *p* > 0.050
Kelly et al. (32) Gluten-free diet	–	BPRS: -No change	SANS: -Cohen’s *d* = −0.53
Kelly et al. (33) OEI	–	BPRS: -Pre-intervention: 14.4 -Post-intervention: 12.2 Mean difference = −2.2 *p* = not statistically significant	SANS: -No change between pre- and post-intervention
Roffman et al. (34) Folic acid and vitamin B12	Total score on PANNS: -Control: −0.22 -Experimental: −0.21 Mean difference = 0.01 *p* = 0.89	PANSS: -Control: -0.04 -Experimental: -0.06 Mean difference = −0.02 *p* = 0.61	SANS: -Control: 0.02 -Experimental: -0.19 Mean difference = −0.21 *p* = 0.15
Neriman et al. (36) Vitamin D	–	SAPS: -Pre-intervention: 18.58 -Post-intervention: 15.51 *p* < 0.001*	SANS: -Pre-intervention: 51.51 -Post-intervention: 23.6 *p* < 0.001*
Pawelckyz et al. (37) Fish oil	–	Correlation between triglyceride levels and total PANSS *p* = 0.031*	Correlation between triglyceride levels and the general psychopathology subscale of PANSS *p* = 0.0008*
Qiao et al. (38) Fish oil	Total score on PANNS: -Control: 59.91 -Experimental: 67.38 Mean difference = +7.47 *p* = 0.19	PANSS: -Control: 16.00 -Experimental: 17.17 Mean difference = 1.17 *p* = 0.27	PANSS: -Control: 14.65 -Experimental: 16.63 Mean difference = 1.98 *p* = 0.05*
Qiao et al. (39) Fish oil	Total score on PANNS: -Control: 63.00 -Experimental: 61.65 Mean difference = −1.35 *p* = 0.86	PANSS: No significant difference between groups; both groups decreased from baseline.	PANSS: No significant difference between groups; both groups decreased from baseline.
Rensburg et al. (40) Ethyl-EPA	Correlation between symptom improvement and PUFA concentrations in the experimental group with >20% improvement on the total PANSS score	Correlation between symptom improvement and PUFA concentrations in the experimental group with >20% improvement on the positive symptom PANSS score	Correlation between symptom improvement and PUFA concentrations in the experimental group with >20% improvement on the negative symptom PANSS score
Vaughan and McConaghy (43) Megavitamin	BDI and BSI: No significant difference	–	–

*PANSS*, Positive and Negative Syndrome Scale; *BPRS*, Brief Psychiatric Rating Scale; *SANS*, Scale for the Assessment of Negative Symptoms; *SAPS*, Scale for the Assessment of Positive Symptoms; *BDI*, Beck’s Depression Inventory; *BSI*, Brief Symptom Inventory; *PUFA*, polyunsaturated fatty acid.

* indicates that the p-value was statistically significant.

## 4 Discussion

Current management of schizophrenia includes administration of antipsychotics, which are well known for increasing the risk of metabolic syndrome. Dysregulation of the inflammatory pathways seen in patients with schizophrenia might play a potential role in its pathogenesis and the subsequent development of metabolic syndrome often seen in these patients. Mental illnesses such as depression, anxiety, and bipolar disorder contribute heavily to global disability; however, the current treatments address less than half of this burden, highlighting the need for complementary strategies. Nutritional psychiatry suggests that the diet quality could be a modifiable risk factor, with evidence linking diet to mental health via pathways such as inflammation, oxidative stress, the gut microbiome, and neuroplasticity (8). An analysis of systematic reviews found that dietary interventions combined with lifestyle therapies can help reduce body weight, BMI, and waist circumference in people with schizophrenia on antipsychotic medications; however, the effects on glycemic control, blood pressure, and triglycerides have been mixed (31). This evidence was rated as suggestive to weak, highlighting the need for higher-quality research and standardized reporting to better understand effective intervention elements and support future dietetic practice and policy (31). Recent research has suggested that lifestyle modifications may be equally, if not more, impactful as a pharmacological treatment for reducing the risk of the metabolic side effects of antipsychotics (15). Although the exact pathogenesis is not well defined, increased inflammation may play an important role in schizophrenia. Specialized diets might not only improve the metabolic outcomes but also reduce inflammation and improve symptoms in people with schizophrenia.

A systematic review in 2017 of RCTs found that adjunctive vitamin B supplementation, particularly B6, B8, and B12, significantly reduced psychiatric symptoms in people with schizophrenia compared with placebo, although effects on the specific symptom domains (positive and negative) were not observed (47). Notably, a shorter illness duration correlated with greater effectiveness of vitamin B, while other supplements, such as antioxidant vitamins and minerals, showed no significant impact (47). These findings suggest that targeted nutritional supplementation could offer symptom relief in schizophrenia, although further research is needed to refine the nutrient formulations and explore the underlying mechanisms (47). Similarly, a review of studies up to 2015 found stronger support for fatty acid supplementation in improving psychotic symptoms and reducing extrapyramidal side effects; however, limitations such as a small sample size and a short study duration highlighted the need for larger, longer-term studies focused on varied patient profiles and cognitive outcomes (48). Thus, our review analyzing the effects of anti-inflammatory diets and supplementation makes strides in addressing the knowledge gap by incorporating more recent studies and analyzing both metabolic and psychiatric symptoms.

Our systematic review revealed mixed findings regarding whether anti-inflammatory diets and supplementation of omega-3 fatty acids, fish oil, and vitamin D are effective in the treatment of metabolic markers or schizophrenia symptoms. The results across multiple studies with similar interventions had various degrees of statistical significance. People with schizophrenia have decreased nutritional intake, including a higher intake of animal fats compared with vegetable fats and instant meals and a lower intake of fruits (22–24). This underlying poor dietary pattern might generate higher baseline inflammation levels that could counteract the potential benefits from targeted interventions.

### 4.1 Strengths and limitations

This review has several important methodological strengths. Firstly, a comprehensive search was conducted across multiple databases, including unpublished literature, to minimize publication bias. Secondly, both dietary and supplementation interventions were evaluated, allowing for broader insights into anti-inflammatory approaches. Thirdly, both metabolic and psychiatric outcomes were assessed, where available, providing a more complete picture of the intervention effects.

However, several limitations affected the interpretation of our findings. Firstly, most of the included studies had small sample sizes and relatively short intervention periods, potentially insufficient to detect meaningful clinical changes. Secondly, there was poor standardization of the interventions, with varying supplement doses and dietary protocols, making direct comparisons challenging. Thirdly, most of the studies failed to measure intervention adherence, particularly in outpatient settings. Fourthly, only a few studies controlled for baseline dietary intake or measured both metabolic and psychiatric outcomes. Finally, there was minimal assessment of cost-effectiveness, which limits our understanding of real-world feasibility.

### 4.2 Implementation challenges and future directions

The implementation of dietary interventions faces significant practical challenges. People with schizophrenia often experience external barriers, including limited financial resources, inadequate social support, and impaired decision-making capacity (49). Internal barriers such as emotional eating and entrenched dietary habits further complicate the adoption of lifestyle changes (49). A 2013 systematic review identified key drivers of medication non-adherence in patients with schizophrenia, such as lack of insight, medication beliefs, and substance abuse, which contribute to negative outcomes such as increased hospitalization and relapse rates. While medication non-adherence and lifestyle intervention and supplementation non-adherence cannot be compared, the barriers to medication adherence might inform the challenges in the implementation of other treatments (50). Another study identified several modifiable barriers to healthy eating for individuals with serious mental illnesses, including both internal factors (e.g., taste preferences, comfort eating, and prioritization of mental health) and external factors (e.g., limited access to healthy foods, social pressures, and medication side effects) (7). To support dietary improvements and to reduce cardiovascular risks, the recommended interventions included individualized nutrition education, opportunities for hands-on healthy food preparation, and involving family and friends. The authors of the aforementioned study additionally recommended that community mental health centers and group homes should offer only healthy foods and that practitioners should discuss emotional eating and the dietary effects of psychiatric medications with patients (7). While there is limited evidence of nutrition interventions significantly improving metabolic syndrome risk factors in people with serious mental illness, one study found that interventions showed more effectiveness when delivered individually or by dietitians. Integrating dietitian-led, personalized dietary programs within mental health services—using behavior change techniques, goal setting, and self-monitoring alongside peer support—could enhance adherence, reduce attrition, and potentially yield cost savings for healthcare systems (31). A recent scoping review found that only 25% of dietary intervention studies in mental health populations demonstrated clear cost-effectiveness (51).

These findings underscore the need for more comprehensive studies that ensure that effective, affordable dietary interventions can be widely implemented, particularly in low-resource settings where the burden of mental illness is highest. Future studies into the impact of anti-inflammatory diets should target larger sample sizes, longer duration of interventions, and more standardized dietary interventions. Supplementation alone may not be adequate to combat the inflammatory disruption associated with either metabolic syndrome or schizophrenia. Future dietary interventions may need to combine an anti-inflammatory diet with other lifestyle changes and/or dietary supplementation to sufficiently impact metabolic markers and improve schizophrenia symptoms.

## 5 Conclusion

The present systematic review reveals important insights into the potential role of anti-inflammatory supplements and diets in schizophrenia while highlighting specific gaps in current research. The mixed findings across studies reflect both the complexity of the implementation of dietary interventions in this population and the challenges of the current research methodology. While some interventions, particularly supplementation with fish oil and vitamin D, showed promise in symptom reduction—and prebiotics/probiotics demonstrated metabolic benefits—the overall evidence remains inconclusive due to methodological limitations.

## Data Availability

The original contributions presented in the study are included in the article/**Supplementary Material**. Further inquiries can be directed to the corresponding author.
