# Supplementary material for: The role of anti-inflammatory diets and supplementation in metabolic syndrome and symptom remission in adults with schizophrenia: a systematic review
Source: Front Psychiatry. 2025 Jan 7;15:1506353. doi: 10.3389/fpsyt.2024.1506353 (PMC11747649; doi:10.3389/fpsyt.2024.1506353)
Supplement: Supplementary file 1 [file Table1.docx]

**Supplementary Table 1.** Strategies for database searches (date of inception through October 24^th^, 2024).

| **Database** | **Search strategy** | **Hits** |
| --- | --- | --- |
| **PubMed/Medline (including Pre-Medline and non-Medline)** | (((((plant-based diet [tw] OR diet [tw] OR anti-inflammatory diet [tw] OR nutrition therapy [tw] OR ketogenic diet [tw] OR keto OR mediterranean diet [tw] OR DASH diet [tw] OR vegetarian diet [tw] OR diet therapy [tw]) AND (first episode psychosis [tw] OR schizophrenia OR psychotic disorders [tw] OR psychosis OR schizophreniform [tw] OR mental disease [tw] OR mental illness [tw] OR brief psychotic disorder [tw] OR schizoaffective disorder [tw] OR schizophreniform disorder [tw] OR schizophrenia spectrum disorder [tw])) NOT (anxiety OR depression OR guilt OR worry OR personality disorder [tw] OR stress OR trauma OR substance use [tw] OR mania OR eating disorder [tw] OR anorexia OR bulimia OR alcoholism OR autism OR Alzheimer disease [tw] OR substance abuse [tw] OR bipolar disorder [tw] OR mood disorders [tw] OR diabetes OR smoking OR post traumatic stress disorder [tw] OR dementia OR PTSD OR obesity)) NOT ((chapter OR book chapter [tw] OR editorial OR commentary OR letter OR review OR survey OR note OR opinion OR systematic review [tw] OR conference abstract [tw] OR case report [tw])) NOT ("animals"[mesh] NOT "humans"[mesh]) AND (english[Filter])) | **245** |
| **Scopus (Elsevier)** | ( TITLE-ABS-KEY ( ( "plant-based diet" OR diet OR "anti-inflammatory diet" OR "nutrition therapy" OR "ketogenic diet" OR keto OR "mediterranean diet" OR "DASH diet" OR "vegetarian diet" OR "diet therapy" ) ) AND TITLE-ABS-KEY ( ( "first episode psychosis" OR schizophrenia OR "psychotic disorders" OR psychosis OR schizophreniform OR "mental disease" OR "mental illness" OR "brief psychotic disorder" OR "schizoaffective disorder" OR "schizophreniform disorder" OR "schizophrenia spectrum disorder" ) ) AND NOT TITLE-ABS-KEY ( ( anxiety OR depression OR guilt OR worry OR "personality disorder" OR stress OR trauma OR "substance use" OR mania OR "eating disorder" OR anorexia OR bulimia OR alcoholism OR autism OR "Alzheimer disease" OR diabetes OR "bipolar disorder" OR obesity OR ptsd OR "post traumatic stress disorder" OR smoking OR "substance abuse" OR "mood disorder" ) ) AND NOT TITLE-ABS-KEY ( ( "case report" OR chapter OR "book chapter" OR editorial OR commentary OR letter OR review OR survey OR note OR opinion OR "systematic review" OR "conference abstract" ) ) AND NOT TITLE-ABS-KEY ( ( animals OR "animal studies" ) ) ) AND ( LIMIT-TO ( DOCTYPE , "ar" ) ) AND ( LIMIT-TO ( LANGUAGE , "English" ) ) | **592** |
| **Embase** | #1 ‘diet'/exp OR 'inflammatory diet' OR 'diet therapy'/de OR 'ketogenic diet'/exp OR 'Mediterranean diet'/exp OR 'DASH diet'/de OR 'vegetarian diet'/exp OR 'plant-based diet' OR 'nutrition therapy'  #2 ‘psychosis'/exp OR 'first episode psychosis' OR 'schizophrenia'/exp OR 'mental disease'/de OR 'schizophreniform disorder'/de OR 'schizophrenia spectrum disorder'/exp OR 'psychotic disorders' OR 'mental illness' OR 'brief psychotic disorder' OR 'schizoaffective psychosis'  #3 #1 AND #2  #4 ‘depression'/exp OR 'anxiety'/exp OR 'guilt'/exp OR 'worry' OR 'personality disorder'/exp OR 'physiological stress'/exp OR 'trauma' OR 'mood disorder'/exp OR 'bipolar disorder'/exp OR 'mania'/de OR 'eating disorder'/exp OR 'bulimia'/de OR 'anorexia nervosa'/de OR 'anorexia' OR 'diabetes mellitus'/exp OR 'substance use'/exp OR 'substance abuse'/de OR 'dementia'/exp OR 'Alzheimer disease'/de OR 'autism'/exp OR 'alcoholism'/de OR 'smoking'/exp OR 'posttraumatic stress disorder'/exp OR 'body weight disorder'/exp OR 'obesity'/exp  #5 'chapter' OR 'book chapter' OR 'letter'/exp OR 'editorial'/de OR 'commentary' OR 'review'/exp OR 'systematic review'/de OR 'note'/de OR 'case report'/de OR 'conference paper'/exp OR 'conference abstract'/de OR 'opinion' OR 'survey'  #6 (‘animal'/exp OR 'animal experiment'/exp OR 'nonhuman') NOT ('human'/exp OR 'human experiment'/exp)  #3 NOT #4 NOT #5 NOT #6 AND english]/lim | **492** |
| **Cochrane Central Register of Controlled Trials (CENTRAL) (Wiley)** | Title abstract Keyword: ("first episode psychosis" OR schizophrenia OR "psychotic disorders" OR psychosis OR schizophreniform OR "mental disease" OR "mental illness" OR "brief psychotic disorder" OR "schizoaffective disorder" OR "schizophreniform disorder" OR "schizophrenia spectrum disorder") NOT (anxiety OR depression OR guilt OR worry OR "personality disorder" OR stress OR trauma OR "substance use" OR mania OR "eating disorder" OR anorexia OR bulimia OR alcoholism OR autism OR "Alzheimer disease" OR diabetes OR "bipolar disorder" OR obesity OR ptsd OR "post traumatic stress disorder" OR smoking OR "substance abuse" OR "mood disorder") AND ("plant-based diet" OR diet OR "anti-inflammatory diet" OR "nutrition therapy" OR "ketogenic diet" OR keto OR "mediterranean diet" OR "DASH diet" OR "vegetarian diet" OR "diet therapy") | **181** |
| **PsycINFO (Ebsco)** | ((plant-based diet OR diet OR anti-inflammatory diet OR nutrition therapy OR ketogenic diet OR keto OR mediterranean diet OR DASH diet OR vegetarian OR diet therapy) ) AND ( (first episode psychosis OR schizophrenia OR psychotic disorders OR psychosis OR schizophreniform OR mental disease OR mental illness OR brief psychotic disorder OR schizoaffective disorder OR schizophreniform disorder OR schizophrenia spectrum disorder)) NOT ((anxiety OR depression OR guilt OR worry OR personality disorder OR stress OR trauma OR substance use OR substance abuse OR mania OR eating disorder OR anorexia OR bulimia OR autism OR dementia OR diabetes OR alcoholism OR bipolar disorder OR mood disorder OR post traumatic stress disorder OR obesity OR alzheimer disease OR PTSD)) NOT ((animals OR animal studies)) NOT ((case report OR book chapter OR chapter OR review OR systematic review OR survey OR letter OR commentary OR note OR editorial OR opinion OR conference abstract OR conference proceeding)) | **227** |
| **ClinicalTrials.gov** | **Condition or Disease:** ("first episode psychosis" OR schizophrenia OR "psychotic disorders" OR psychosis OR schizophreniform OR "mental disease" OR "mental illness" OR "brief psychotic disorder" OR "schizoaffective disorder" OR "schizophreniform disorder" OR "schizophrenia spectrum disorder") NOT (anxiety OR depression OR guilt OR worry OR "personality disorder" OR stress OR trauma OR "substance use" OR mania OR "eating disorder" OR anorexia OR bulimia OR alcoholism OR autism OR "Alzheimer disease" OR diabetes OR "bipolar disorder" OR obesity OR ptsd OR "post traumatic stress disorder" OR smoking OR "substance abuse" OR "mood disorder")  **Intervention/treatment:** ("plant-based diet" OR diet OR "anti-inflammatory diet" OR "nutrition therapy" OR "ketogenic diet" OR keto OR "mediterranean diet" OR "DASH diet" OR "vegetarian diet" OR "diet therapy")  **Other terms**: NOT(animals) | **240** |
| **ISRCTN Registry** | ("first episode psychosis" OR schizophrenia OR "psychotic disorders" OR psychosis OR schizophreniform OR "mental disease" OR "mental illness" OR "brief psychotic disorder" OR "schizoaffective disorder" OR "schizophreniform disorder" OR "schizophrenia spectrum disorder") AND ("plant-based diet" OR diet OR "anti-inflammatory diet" OR "nutrition therapy" OR "ketogenic diet" OR keto OR "mediterranean diet" OR "DASH diet" OR "vegetarian diet" OR "diet therapy" | **132** |
| **ICTRP** | ("first episode psychosis" OR schizophrenia OR "psychotic disorders" OR psychosis OR schizophreniform OR "mental disease" OR "mental illness" OR "brief psychotic disorder" OR "schizoaffective disorder" OR "schizophreniform disorder" OR "schizophrenia spectrum disorder") AND (("plant-based diet" OR diet OR "anti-inflammatory diet" OR "nutrition therapy" OR "ketogenic diet" OR keto OR "mediterranean diet" OR "DASH diet" OR "vegetarian diet" OR "diet therapy") | **14** |
|  |  |  |
